# Impact of Early Mobilization and Rehabilitation on Functional Outcomes Following Craniocerebral Gunshot Injuries

**DOI:** 10.1002/ccr3.72645

**Published:** 2026-05-01

**Authors:** Md. Abdul Alim, Kazi Md Azman Hossain, Sabrina Tina, Mahmuda Akter Akhi, Azharul Islam

**Affiliations:** ^1^ Department of Physiotherapy Centre for the Rehabilitation of the Paralysed (CRP) Dhaka Bangladesh; ^2^ Department of Physiotherapy and Rehabilitation Jashore University of Science and Technology (JUST) Jashore Bangladesh; ^3^ Department of Physiotherapy Bangladesh Health Professions Institute (BHPI) Dhaka Bangladesh

**Keywords:** craniocerebral injuries, early mobilization, gunshot injuries, rehabilitation, traumatic brain injuries

## Abstract

Craniocerebral gunshot injury (CGI) is a severe form of traumatic brain injury associated with profound neurological, cognitive, and functional impairment, often requiring prolonged rehabilitation. We report the case of a 17‐year‐old boy with a penetrating CGI who presented with a Glasgow Coma Scale score of 3/15, intracranial hemorrhage, a depressed frontal skull fracture, retained bullet fragments, and complete dependence in activities of daily living. Following neurosurgical stabilization, the patient underwent a structured, multidisciplinary neurorehabilitation program over 12 weeks (48 sessions). The intervention focused on pain management, spasticity control, postural and trunk stabilization, balance and gait training, cognitive stimulation, and task‐oriented functional practice. Post‐intervention assessments indicated resolution of pain, reduction in muscle tone abnormalities, improved functional independence, progression to assisted ambulation, enhanced cognitive function, and improved health‐related quality of life. These findings suggest that structured multidisciplinary neurorehabilitation may be associated with functional improvements after CGI.

## Key Clinical Message

Early, structured, multidisciplinary neurorehabilitation was associated with improvements in independence, mobility, cognition, and quality of life in an adolescent after craniocerebral gunshot injury. Progressive intervention, task‐specific training, and coordinated care may support neurological recovery and community reintegration.

## 1 Introduction

Craniocerebral gunshot injuries (CGIs) constitute a critical and escalating public health problem, particularly in civilian and urban settings of developing countries where sociopolitical unrest and armed violence persist. Although less prevalent than closed head trauma, penetrating brain injury carries a markedly worse prognosis [1]. CGI is considered the most lethal of all firearm‐related injuries, with reported survival rates of only 7% to 15%. Approximately 90% of victims die before reaching hospital care, and among those who arrive alive, nearly 50% die in the emergency room [1, 2, 3]. Mortality is highest either at the site of injury or within the first 3 h post‐trauma, underscoring the narrow therapeutic window for life‐saving intervention [1, 4, 5].

These injuries place substantial demands on neurosurgical services and already strained healthcare systems. Optimal management requires rapid, coordinated, and evidence‐based decision‐making. Initial care emphasizes aggressive resuscitation, stabilization of airway, breathing, and circulation, and prompt correction of coagulopathy [1, 6]. In patients with stable hemodynamics, early head computed tomography (CT) is indispensable for diagnostic clarification and operative planning. Neuroimaging guides the determination of the surgical approach, extent of debridement, and localization of retained foreign bodies. While operative management is frequently indicated, selected cases may be managed conservatively based on neurological status and radiological findings [6, 7].

Beyond immediate survival, the pathophysiology of CGI further complicates prognosis. High‐velocity projectiles generate extensive cavitation, secondary bone fragmentation, and diffuse neuronal injury, amplifying both primary and secondary brain damage [8]. Elevated intracranial pressure (ICP) remains a leading cause of secondary deterioration, and failure to control ICP is strongly associated with poor neurological outcomes [9]. Even among surgically managed patients, mortality rates have been reported to range from 7.4% to 18.7% in recent studies, highlighting the persistent severity of these injuries [1].

However, survival alone does not define successful management. Functional recovery and quality of life are increasingly recognized as critical endpoints. Evidence supports a multidisciplinary approach in which early rehabilitation is integrated into acute neurocritical care [10, 11]. Coordinated input from physiotherapists and occupational therapists has been shown to improve functional outcomes in patients with penetrating brain injury. Early mobilization and structured physiotherapy interventions reduce secondary complications such as joint contractures, muscle atrophy, and pulmonary dysfunction, while promoting improvements in motor control, balance, and muscular strength [12, 13]. Given the high burden of neurological impairment among survivors, timely rehabilitation may significantly influence long‐term independence and societal reintegration.

In countries such as Bangladesh, where access to specialized trauma and neurorehabilitation services remains limited, the integration of early mobilization strategies into standard CGI management is particularly crucial. This case report describes the rehabilitation trajectory of a 17‐year‐old boy who sustained a penetrating CGI during the anti‐discrimination movement and outlines the observed functional changes associated with early mobilization and a structured rehabilitation program in a resource‐constrained clinical setting.

## 2 Case Description

**FIGURE 1:**
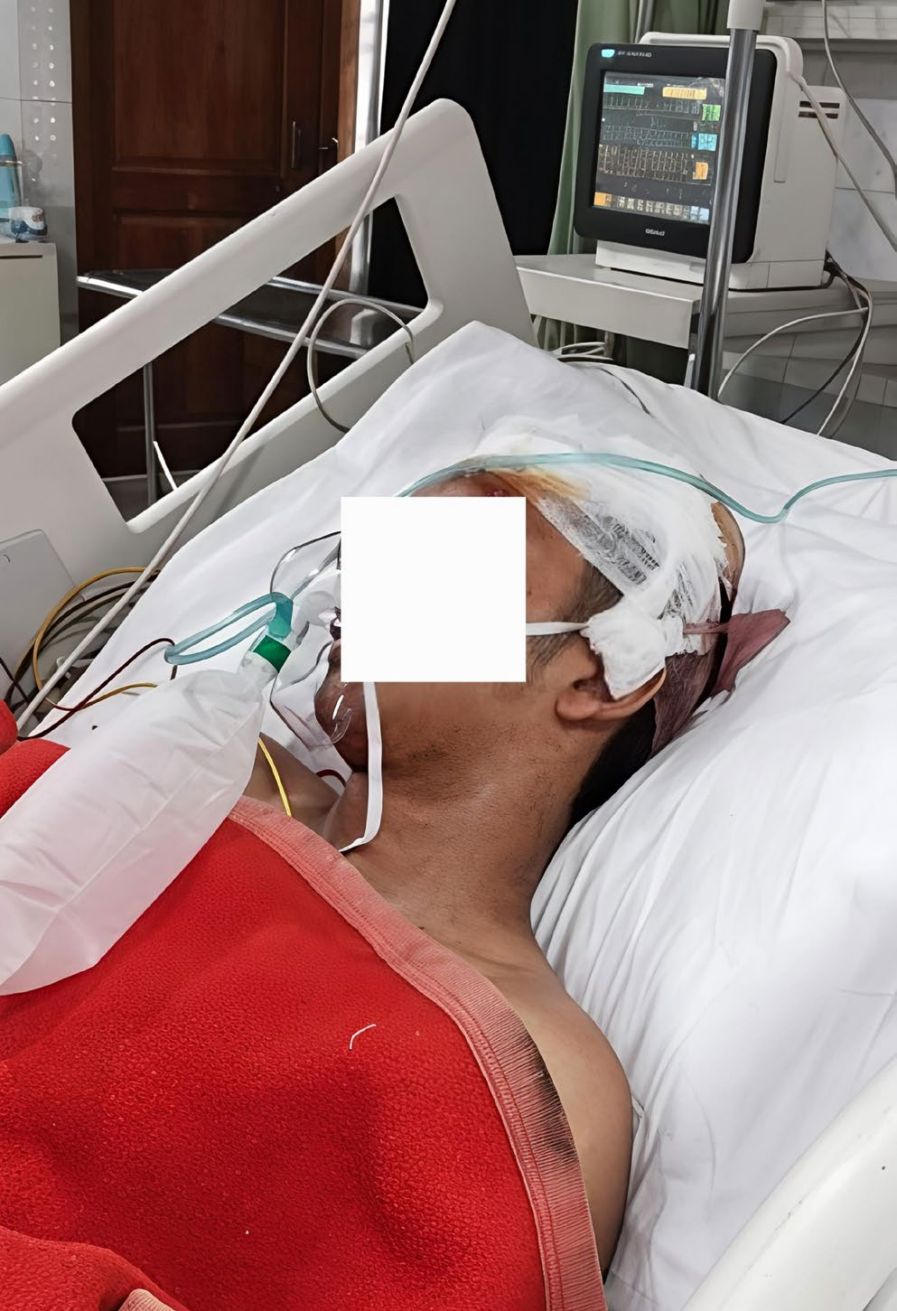
Patient receiving neurological treatment during hospitalization.

In 2024, a 17‐year‐old male student sustained a severe CGI during a nationwide anti‐discrimination movement (Figure 1). He had no prior history of chronic medical illness, including diabetes mellitus, hypertension, cardiovascular disease, or respiratory disorders. Immediately following the injury, he was transported to a local healthcare facility, where emergency stabilization was initiated. Given the severity of the head trauma, he was referred to a tertiary‐level hospital for definitive management. Upon arrival, he was deeply unconscious, with a Glasgow Coma Scale (GCS) score of 3/15. Pupillary examination demonstrated bilaterally equal and reactive pupils. Airway protection was promptly achieved through endotracheal intubation. An urgent non‐contrast computed tomography (CT) scan of the brain revealed acute intracranial hemorrhage with subarachnoid extension, a depressed frontal skull fracture, and retained intracranial bullet fragments along the projectile trajectory, without an identifiable exit wound. Multiple bone fragments were also observed along the bullet path.

**FIGURE 2:**
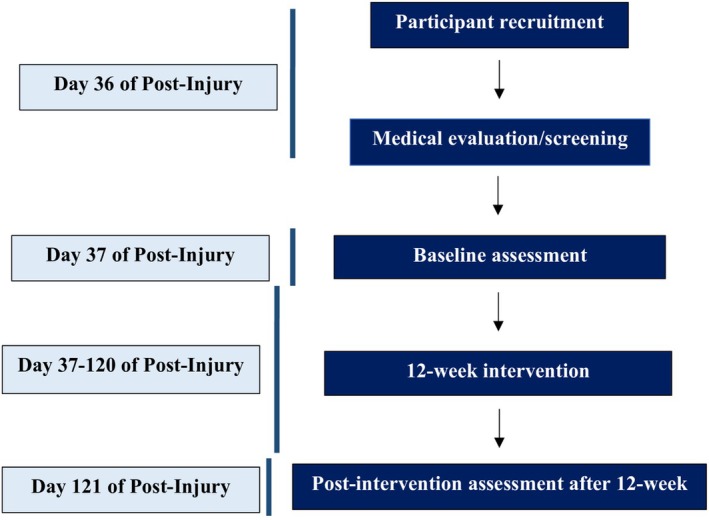
Study flow diagram.

The patient was admitted to the intensive care unit (ICU) on the first day and managed with comprehensive neurocritical care, including mechanical ventilation, intracranial pressure–directed medical therapy, osmotherapy for cerebral edema, anticonvulsant prophylaxis, and broad‐spectrum antibiotics to reduce the risk of intracranial infection. He remained on mechanical ventilation for 11 days, during which serial neurological assessments were performed. Due to the complexity of the injury and the presence of retained intracranial foreign bodies, he was subsequently transferred via air ambulance on Day 12 to the National Institute of Neurosciences. Following multidisciplinary evaluation, a frontal craniotomy was performed for targeted debridement of necrotic brain tissue, removal of retained bullet and bone fragments, evacuation of intracranial hematoma, meticulous hemostasis, and dural repair on Day 13–Day 14. Postoperatively, the patient received continued neuro–ICU care, with close monitoring for infection, cerebral protection strategies, and ventilatory support. Mechanical ventilation was successfully weaned following neurological stabilization, and the patient's GCS improved to 10/15 by Day 21. After achieving medical stability, a 10‐day period of supervised bed rest was implemented to support cerebral recovery. He was subsequently referred to the Centre for the Rehabilitation of the Paralyzed (CRP) for comprehensive multidisciplinary rehabilitation. And he came to CRP for rehabilitation on the 36th day of his post‐injury period (Figure 2).

## 3 Examination and Evaluation

Upon admission to CRP, the patient underwent a detailed neurological, musculoskeletal, cognitive, and functional assessment. Neurological examination revealed right‐sided spastic hemiplegia with contralateral lower‐limb weakness. The patient demonstrated intact sensory function, with preserved light‐touch and pinprick perception in all tested regions. The patient reported severe pain, rated 7/10 on the Visual Analog Scale (VAS), which significantly limited active participation in therapy and restricted functional mobility. Spasticity was pronounced, with Modified Ashworth Scale (MAS) scores of 3+ in the affected upper limb and 3 in the lower limb.

Postural control was markedly impaired, with poor trunk stability and compromised static and dynamic balance during sitting. The active range of motion was reduced across multiple joints, further limiting functional performance. Functional status, assessed using the Functional Independence Measure (FIM), demonstrated maximum assistance (38/126), and the patient remained non‐ambulatory at baseline.

Cognitive evaluation using the Montreal Cognitive Assessment (MoCA) revealed moderate cognitive impairment (12/30), adversely affecting attention, executive function, learning capacity, and safety during transfers and task engagement. These deficits posed significant challenges to early rehabilitation participation and functional skill acquisition.

Health‐related quality of life was assessed using the Short Form‐36 (SF‐36), which demonstrated profound limitations across all domains: physical functioning (30/100), role physical (25/100), bodily pain (35/100), general health (40/100), vitality (35/100), social functioning (30/100), role emotional (28/100), and mental health (38/100).

Collectively, baseline assessments underscored the need for an intensive, structured, and multidisciplinary rehabilitation program, with prioritized goals including pain management, reduction of spasticity, restoration of functional mobility, cognitive rehabilitation, and improvement in overall quality of life. Although the baseline examination and evaluation were slightly challenging due to the patient's cognitive impairments, we addressed these issues by using repetitive, clear, and technical commands and procedures.

## 4 Intervention

A structured, evidence‐based, neurorehabilitation program was initiated immediately upon admission, following a comprehensive clinical assessment and individualized goal setting. The primary objectives were to prevent secondary complications (e.g., joint contractures, disuse atrophy, and cardiopulmonary deconditioning), reduce pain, normalize muscle tone, restore joint mobility, improve muscle strength and sensory feedback, and enhance functional mobility and independence. The program was implemented over 12 weeks, comprising 48 supervised sessions, and followed a structured, outcome‐driven progression model. In the early phase, treatment emphasized pain management, passive and active‐assisted range‐of‐motion exercises, chest physiotherapy, early mobilization, bed mobility training, and facilitation of trunk control to promote neuromotor activation and prevent immobility‐related complications [10, 14, 15, 16].

As neurological stability improved, the intervention expanded to include muscle tone modulation and motor‐recovery strategies such as stretching, proprioceptive neuromuscular facilitation, neuromuscular electrical stimulation, and joint mobilization, alongside progressive postural control and balance training using static and dynamic tasks. Vestibular rehabilitation and sensory integration exercises were incorporated to address impairments in spatial orientation and cognitive–motor coordination. In the later stages, the focus shifted to neuromuscular re‐education and task‐specific functional training, including sit‐to‐stand practice, transfer training, activities of daily living simulation, coordination exercises, assisted gait training, and progressive strengthening and endurance conditioning. Treatment intensity and complexity were continuously adjusted based on clinical response and functional milestones to ensure safe progression and optimize outcomes [10, 16, 17]. Psychological support and counseling were also incorporated into patient education as part of holistic rehabilitation in regular sessions to address emotional distress, trauma‐related responses, and adjustment challenges.

A detailed description of intervention components and progression is provided in Table 1.

**TABLE 1:** Intervention plan for 12 weeks.

Intervention	Techniques/exercises	Dosage	Patient status addressed	Post‐intervention goal/justification
Early mobilization	Passive ROM, Active‐assisted movements, Bed mobility, Chest physiotherapy	10–15 reps/joint; 10–15 min mobility; low intensity (RPE 1–2); progress to active movement when pain ≤ 3 (VAS) and voluntary control emerges	Severe pain, immobility, risk of contractures	Pain reduction (VAS 0), initiate neuro‐motor recovery, prevent secondary complications, enable active rehab participation
Muscle tone management	MAS‐guided stretching, PNF, NMES, Joint mobilization	Stretch 20–30 s × 3–5; NMES 15–20 min; low–moderate intensity; progress with MAS ↓ ≥ 1 grade	Spasticity limiting transfers, reaching, and standing	Normalize muscle tone, improve voluntary movement, enable safe transfers and gait, support functional independence
Postural control and balance	Static and dynamic balance, trunk rotations, head turns, reaching, ball catching	10–20 min; RPE 2–5; progress supported → dynamic → dual‐task	Trunk instability, impaired balance	Improved trunk stability, safe mobility, enhanced dynamic balance, facilitating assisted ambulation, and functional independence
Vestibular and sensory integration	Head‐eye coordination, visual‐vestibular exercises, and sensory integration	10–15 min; symptom‐limited; progress with ↓ dizziness and ↑ tolerance	Dizziness, spatial disorientation, impaired cognitive‐motor coordination	Reduce dizziness, improve spatial orientation, support cognitive engagement, enhance safety and participation in rehab
Neuromuscular facilitation	NMES, Manual therapy, Stretching	NMES 15–20 min; low–moderate; progress with ↑ voluntary control	Spasticity, weakness	Facilitate muscle reeducation, reduce tone, improve voluntary control, and ADL performance
Task‐specific and functional training	Sit‐to‐stand, transfers, ADL simulations, assisted gait, coordination tasks	2–3 sets ×8–12 reps; 10–15 min gait; RPE 3–6; progress assisted → independent	Dependence in ADLs and transfers	Independence, improved mobility, safe ADL execution, functional recovery, QoL gains
Strength and endurance	Core exercises, limb strengthening, theraband/weight exercises	2–3 sets ×10–15 reps; 10–20 min; RPE 3–6; progress when ≥ 15 reps tolerated	Weakness, poor endurance	Improved muscular support, functional mobility, activity tolerance, and QoL
Progression and Individualization	Stepwise intensity increase, tolerance‐based pacing, daily monitoring	60–90 min/session; progression based on VAS ≤ 2–3, MAS ↓, FIM ↑, fatigue tolerance	Fatigue, neurological symptom variability	Ensure safe, optimized rehabilitation, and gradual improvement in all outcome measures

*Note:* The patient completed a 12‐week, 48‐session individualized rehabilitation program (4 sessions/week, 60–90 min each). Progression was outcome‐driven, stepwise, and continuously monitored: Weeks 1–4: Focused on pain reduction, passive/active‐assisted ROM, bed mobility, and early trunk control; NMES and MAS‐guided stretching targeted spasticity. Weeks 5–8: Progressed to dynamic balance, sit‐to‐stand, transfers, vestibular exercises, and dual‐task cognitive‐motor training; muscle tone and functional mobility were actively addressed. Weeks 9–12: Advanced task‐specific functional training, assisted ambulation, ADL simulations, strength, endurance, and complex balance exercises; intensity and complexity adjusted to patient tolerance. All interventions were aligned with deficits, continuously individualized, and designed to improve pain, spasticity, mobility, functional independence, cognition, and quality of life, in accordance with current TBI and Berlin Consensus guidelines.

## 5 Outcome Measures

To comprehensively evaluate the efficacy of the structured neurorehabilitation program, a set of standardized, validated, and widely accepted outcome measures was employed. All outcomes were assessed at baseline and immediately following the 12‐week intervention period. The measures spanned six key domains: pain, muscle tone, functional independence, mobility and balance, cognitive function, and health‐related quality of life.

### 5.1 Pain

Pain intensity was measured using the Visual Analogue Scale (VAS), a validated instrument for quantifying subjective pain perception [18]. Participants rated their current pain on a 0–10 scale, with higher scores indicating greater pain. At baseline, pain limited engagement in mobility, transfers, and rehabilitation activities.

### 5.2 Muscle Tone

Muscle tone was assessed using the Modified Ashworth Scale (MAS), a widely recognized clinical tool for evaluating spasticity in both upper and lower limbs [19]. Elevated muscle tone initially restricted voluntary movement and functional use of the limbs.

### 5.3 Functional Independence

Functional capacity was evaluated using the Functional Independence Measure (FIM), which quantifies the level of assistance required for activities of daily living [20]. Initial assessments reflected maximum assistance due to impaired mobility and reduced motor control.

### 5.4 Mobility and Balance

Functional mobility and dynamic balance were assessed using the Timed Up and Go (TUG) test. This test evaluates the ability to stand, walk, turn, and sit safely [21]. Limited baseline mobility and balance, restricted ambulation.

### 5.5 Cognitive Function

Cognitive performance was assessed using the Montreal Cognitive Assessment (MoCA), which covers attention, executive function, memory, language, and orientation [22]. Baseline cognitive limitations affected task comprehension, safety awareness, and adherence to therapy.

### 5.6 Health‐Related Quality of Life

Quality of life was assessed using the Short Form‐36 (SF‐36), capturing physical, emotional, and social health domains [23]. Baseline dependence, limited mobility, and pain negatively impacted daily participation and perceived health.

## 6 Results

Following completion of the 12‐week rehabilitation program, the patient demonstrated substantial improvements across all outcome domains, with no adverse events reported. Pain intensity decreased from 7/10 at baseline to 0/10 post‐intervention, indicating complete pain resolution and removal of a major barrier to active rehabilitation. Spasticity decreased substantially, with MAS scores improving from 3+ to 0 in the upper limbs and from 3 to 0 in the lower limbs, indicating normalized muscle tone, enhanced motor control, and restored voluntary movement.

**FIGURE 3:**
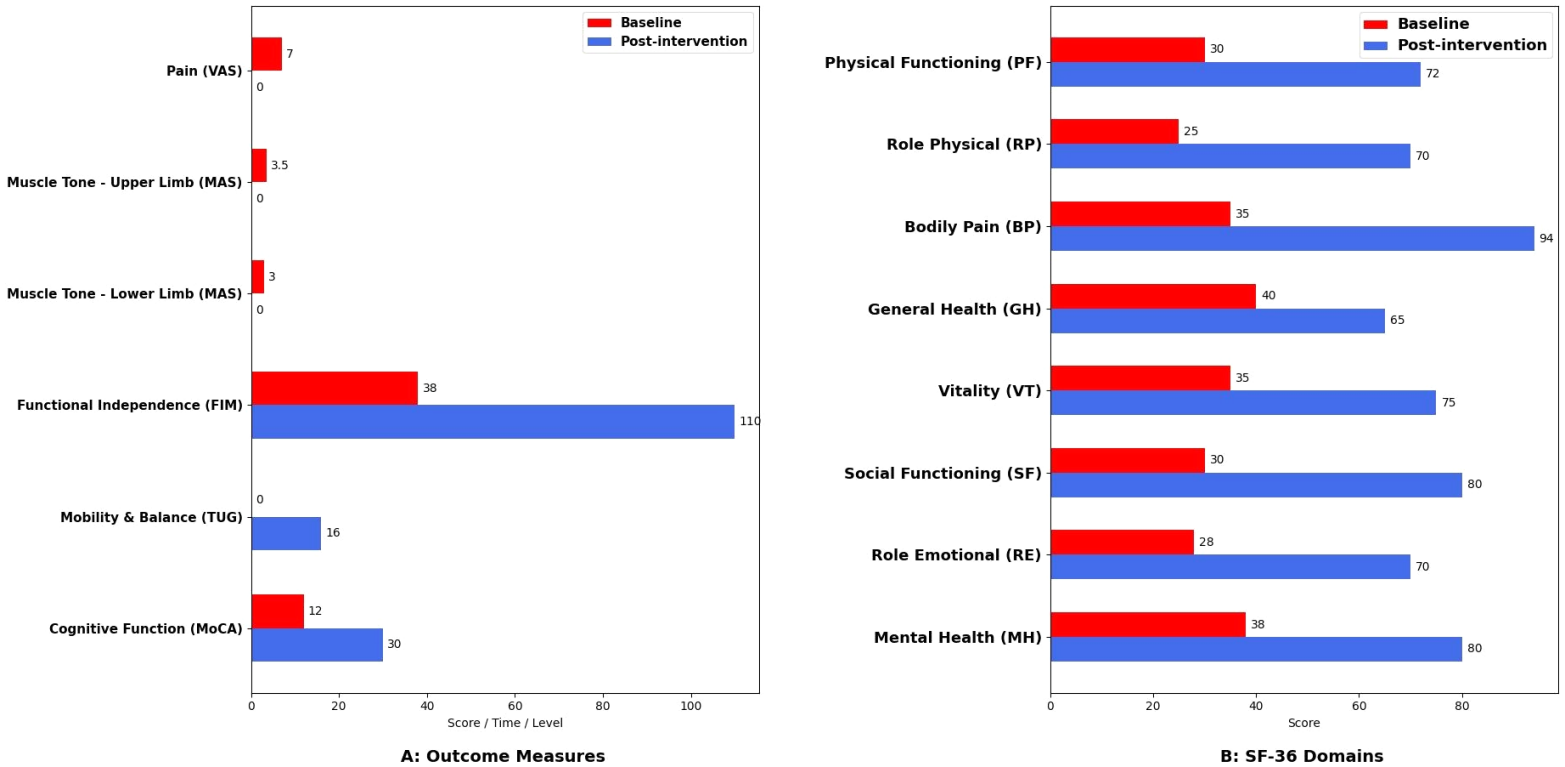
Outcome measures visualization.

FIM scores increased from 38/126 (maximum assistance) to 110/126 (modified independence), demonstrating significant recovery in functional autonomy and the ability to perform activities of daily living with reduced assistance. TUG performance improved from a non‐ambulatory, wheelchair‐bound status to a modified ambulation status, with a post‐intervention time of 16 s. This change reflected enhanced trunk stability, balance, and activity tolerance. MoCA scores increased from 12/30 (moderate impairment) to 30/30 (no impairment), suggesting improved cognitive engagement, task understanding, and safety awareness during functional activities. Significant improvements were observed across all SF‐36 domains. Physical Functioning increased from 30/100 to 72/100, Role Physical from 25/100 to 70/100, and Bodily Pain from 35/100 to 94/100. General Health and Vitality improved to 65/100 and 75/100, respectively. Social Functioning rose from 30/100 to 80/100, Role Emotional from 28/100 to 70/100, and Mental Health from 38/100 to 80/100, reflecting enhanced physical, emotional, and social well‐being. Detailed changes in outcomes are summarized in Tables 2 and 3 and shown in Figure 3.

**TABLE 2:** Outcome measures over time.

Outcome	Baseline score	Post‐intervention score	Clinical interpretation
Pain (VAS)	7/10	0/10	Severe pain initially limited mobility, transfers, and participation in therapy. Complete resolution enabled active rehab, improving muscle tone, functional independence, and overall recovery
Muscle tone—upper limb (MAS)	3+	0	High spasticity in the right upper limb restricted fine motor control and transfers. Reduction facilitated improved voluntary movement, functional independence, and upper‐limb task performance
Muscle tone—lower limb (MAS)	3	0	Spasticity in the lower limb limited standing and ambulation. Reduction allowed safer assisted walking, improved balance, and functional mobility
Functional independence (FIM)	38/126 (maximum assistance)	110/126 (modified independence)	Initial dependence due to spasticity, weakness, pain, and poor trunk control. Gains reflect combined improvements in tone, mobility, cognition, and reduced pain
Mobility and balance (TUG)	Non‐ambulatory/wheelchair‐bound	16 s (modified ambulation)	Baseline immobility reflected severe neurological deficits. Post‐intervention modified ambulation is associated with improved FIM, reduced muscle tone, and enhanced trunk stability
Cognitive function (MoCA)	12/30 (moderate impairment)	30/30 (no impairment)	Cognitive deficits initially limited safe mobility and functional task learning. Improvement enabled better adherence to rehab exercises and safe transfers, supporting FIM and mobility gains

Abbreviations: FIM, functional independence measure; MAS, modified ashworth scale; MoCA, montreal cognitive assessment; TUG, timed up and go test; VAS, visual analog scale.

**TABLE 3:** Overall quality of life status based on the SF‐36.

SF‐36 domain	Baseline score	Post‐intervention score	Clinical interpretation
Physical Functioning (PF)	30/100	72/100	Severe limitation due to immobility and spasticity. Post‐intervention improvement mirrors assisted ambulation, FIM gains, and recovery of trunk control
Role physical (RP)	25/100	70/100	Baseline inability to perform daily roles. Post‐intervention improvement reflects a regained capacity for ADLs and partial participation in school/work‐related activities
Bodily pain (BP)	35/100	94/100	Pain initially restricted rehab participation. Post‐intervention resolution enabled active engagement in exercises and functional mobility, thereby improving overall QoL
General health (GH)	40/100	65/100	Baseline poor perception of health due to hemorrhagic CGI. Post‐intervention improvement reflects confidence in physical recovery and functional ability
Vitality (VT)	35/100	75/100	Baseline fatigue due to ICU stay and neurological compromise. Gains indicate improved endurance, activity tolerance, and participation
Social functioning (SF)	30/100	80/100	Severe social limitations due to immobility and dependence. Post‐intervention improvement reflects increased independence, mobility, and interaction ability
Role emotional (RE)	28/100	70/100	Emotional limitations are initially caused by trauma and dependence. Post‐intervention improvement reflects enhanced coping, self‐efficacy, and engagement in meaningful activities
Mental health (MH)	38/100	80/100	Baseline low mood, anxiety, and frustration. Post‐rehab gains reflect improved autonomy, participation, and overall cognitive and functional recovery

## 7 Discussion

Craniocerebral gunshot injuries (CGIs) represent one of the most severe forms of traumatic brain injury, frequently associated with profound neurological impairment, prolonged dependency, and high mortality among survivors [1]. In the present case, the patient initially demonstrated severe neurological compromise characterized by spastic hemiplegia, impaired cognition, maximum functional assistant dependence, and non‐ambulatory status following surgical management of penetrating brain trauma. The observed recovery trajectory following structured neurorehabilitation may support the critical role of early, multidisciplinary rehabilitation in promoting neurological recovery and restoring functional independence after penetrating brain injury.

Pain reduction was among the earliest and most clinically meaningful improvements, with VAS scores decreasing from severe pain at baseline to complete resolution at the post‐intervention assessment. Previous neurorehabilitation studies have demonstrated that uncontrolled pain following traumatic brain injury negatively affects motor learning, participation, and therapy adherence. Effective pain control through therapeutic exercise, gradual mobilization, and neuromuscular activation facilitates cortical engagement and enables active participation in rehabilitation, thereby accelerating functional recovery [4, 24]. The complete resolution of pain in this case likely contributed to improvements observed across mobility, functional independence, and quality‐of‐life outcomes.

Reductions in spasticity and normalization of muscle tone, as reflected by improvements in MAS scores in both upper and lower limbs, are consistent with evidence that early stretching, task‐oriented movement, and neuromuscular facilitation techniques can reduce hypertonicity and enhance voluntary motor control [7]. Spasticity following brain injury is associated with abnormal reflex activity and impaired motor coordination, often limiting functional use of the limbs [25]. The marked reduction observed in this case enabled improved limb positioning, safer transfers, and initiation of assisted ambulation, supporting findings from prior studies demonstrating that early tone management improves functional outcomes and reduces secondary musculoskeletal complications.

FIM scores improved substantially from maximum assistance to modified independence, indicating meaningful gains in activities of daily living and mobility‐related functions. Comparable neurorehabilitation studies in severe traumatic brain injury populations have shown that structured, goal‐oriented rehabilitation programs emphasizing task‐specific training and progressive mobility can produce significant functional gains even in patients presenting with low initial GCS scores [16, 26]. The improvement observed in this case reflects the cumulative effect of motor recovery, improved cognition, reduced pain, and enhanced postural control, emphasizing the multidimensional nature of recovery following CGI.

Mobility outcomes demonstrated a transition from wheelchair dependence to modified ambulation, as reflected by TUG performance at the post‐intervention assessment. Early mobilization following severe brain injury has been shown to reduce complications associated with prolonged immobility, including muscle atrophy, cardiopulmonary deconditioning, and postural instability. The observed improvement in trunk stability and balance is consistent with evidence supporting task‐specific gait training and progressive strengthening as key contributors to regaining functional mobility in neurological populations [16, 27].

Cognitive recovery, reflected by improvement in MOCA scores from moderate to no impairment, further supports the role of integrated rehabilitation approaches. Cognitive deficits frequently limit rehabilitation engagement and safety awareness after penetrating brain injury. Literature indicates that cognitive stimulation combined with physical rehabilitation enhances neuroplasticity through repeated sensorimotor integration and functional task practice [8, 28]. Improved cognition in this case likely facilitated better adherence to therapeutic instructions and contributed indirectly to functional gains.

Quality‐of‐life outcomes, as assessed by SF‐36 domains, demonstrated substantial improvement across physical, emotional, and social components. Improvements in bodily pain, physical functioning, and social participation mirror findings from prior research indicating that functional independence and mobility recovery strongly influence perceived health status and psychological well‐being after traumatic brain injury [12, 29]. The improvement in mental health and emotional role functioning observed in this case underscores the interdependence of physical recovery and psychosocial reintegration, particularly in adolescent patients recovering from life‐threatening trauma.

Psychological and psychiatric support represents an important, yet often underreported, component of neurorehabilitation following traumatic brain injury. In this case, mental health status was assessed through the mental health domains of the SF‐36, alongside ongoing psychological counseling, patient education, and collaborative therapeutic engagement integrated into routine rehabilitation sessions. Improvements observed in mental health‐related quality‐of‐life measures may reflect the combined influence of structured rehabilitation and supportive psychosocial care. Addressing emotional well‐being, trauma‐related stress, and potential underlying psychological conditions is considered important for optimizing rehabilitation engagement and longer‐term community reintegration [29, 30].

As this report describes a single case, causal relationships between the intervention and recovery cannot be established. The observed improvements likely reflect a multifactorial process, including timely neurosurgical management, spontaneous neurological recovery, and younger age, which is associated with greater neuroplastic potential. The extent of recovery in this case appears notable compared with commonly reported outcomes in severe CGI, particularly in patients with very low initial GCS scores; however, similar recovery patterns have occasionally been described in younger individuals with focal injury and early stabilization. Contributing factors may include preserved brain regions outside the projectile trajectory and the absence of major systemic complications [29, 30]. These findings should be interpreted cautiously, as they may not be generalizable to the broader CGI population, where morbidity and disability remain substantial. This case also demonstrates the feasibility of implementing structured, multidisciplinary neurorehabilitation in a resource‐constrained setting, supporting its integration within the continuum of care following severe brain injury.

## 8 Strengths and Limitations

This case report provides a comprehensive, multidimensional assessment of recovery following CGI, using standardized, validated outcome measures (VAS, MAS, FIM, TUG, MoCA, and SF‐36) to capture neurological, functional, and psychosocial domains. The structured and progressive rehabilitation approach reflects routine clinical practice in a resource‐constrained setting, enhancing its practical applicability. Furthermore, the inclusion of cognitive, functional, and quality‐of‐life outcomes offers a more holistic perspective on recovery beyond survival alone.

However, the findings are limited by the single‐case design, restricting generalizability. The absence of long‐term follow‐up precludes evaluation of sustained outcomes, and the lack of objective neurophysiological or advanced imaging assessments limits insight into the underlying mechanisms of recovery.

## 9 Conclusions

This case report highlights clinically meaningful improvements in functional independence, mobility, cognition, and health‐related quality of life following early, structured, multidisciplinary neurorehabilitation after CGI. Recovery occurred in the context of progressive, task‐specific rehabilitation delivered alongside neurosurgical management in a resource‐constrained setting. However, given the single‐case design, causality cannot be established, and the observed changes are likely attributable to a combination of neurosurgical intervention, spontaneous neurological recovery, younger age with greater neuroplastic potential, and structured rehabilitation. These findings support the feasibility and potential value of integrating early rehabilitation within acute neurosurgical pathways for severe penetrating brain injury, while acknowledging the high variability of outcomes in this population. Further prospective studies with larger cohorts and longitudinal follow‐up are required to better characterize recovery trajectories and develop standardized, evidence‐based rehabilitation protocols for CGI.

## 10 Patient's Perspectives

When I first became aware of my condition, I felt completely overwhelmed and frightened. I was only a school student with hopes of sitting for my exams and building a future, and suddenly, those aspirations felt very distant. I could not move properly; I depended on others for even the simplest daily activities, and I constantly worried that I might never return to a normal life. The early days were not only physically painful but also emotionally exhausting. There were moments of deep uncertainty, where I questioned my strength and my future. However, my rehabilitation team stood by me from the outset. They patiently explained every step of my treatment, encouraged me when I felt weak, and never allowed me to lose hope, even when progress felt slow.

As rehabilitation continued, small improvements began to change everything. Being able to sit without support, move more freely, and take part in daily activities gave me renewed confidence. Each small success felt like a victory and reminded me that recovery was possible. When I started walking in a modified pattern and became more independent, I felt as though I was reclaiming control over my own life. Beyond physical recovery, I became mentally stronger and more optimistic. Rehabilitation helped me rebuild my confidence, reconnect with people around me, and regain confidence in my education and future. Today, I look forward with hope, knowing that careful, dedicated rehabilitation played a critical role in restoring my life.

## 11 Learning Points

- CGI causes severe motor, cognitive, and psychosocial impairments requiring comprehensive rehabilitation, but in this single case, recovery cannot be directly attributed to the intervention
- Improvement was likely multifactorial, involving neurosurgical stabilization, spontaneous recovery, and a younger age associated with higher neuroplastic potential.
- Functional gains appeared greater than typically reported in severe CGI with very low initial GCS, although similar outcomes have been observed in select young patients with focal injury and early stabilization.
- Preservation of uninvolved brain tissue and absence of major systemic complications may have contributed to the observed recovery pattern
- Structured multidisciplinary neurorehabilitation was feasible in this resource‐limited setting; however, findings should be interpreted cautiously and not generalized to all CGI cases.

## Author Contributions

**Md. Abdul Alim:** project administration, resources, supervision, writing – original draft, writing – review and editing. **Kazi Md Azman Hossain:** investigation, methodology, visualization, writing – original draft, writing – review and editing. **Sabrina Tina:** methodology, validation, writing – review and editing. **Mahmuda Akter Akhi:** conceptualization, formal analysis, methodology, writing – review and editing. **Azharul Islam:** investigation, methodology, writing – review and editing.

## Funding

The authors have nothing to report.

## Ethics Statement

Ethical approval was obtained from the Institute of Physiotherapy Rehabilitation and Research of the Bangladesh Physiotherapy Association (BPA) before the submission of this manuscript.

## Consent

Written informed consent was obtained directly from patient(s) for assessment, intervention, and to publish this report in accordance with the journal's patient consent policy.

## Conflicts of Interest

The authors declare no conflicts of interest.

## Data Availability

The authors have nothing to report.
